# Macrolitter dataset from a highly frequented roadway in Nantes, France

**DOI:** 10.1016/j.dib.2022.108237

**Published:** 2022-05-04

**Authors:** Romain Tramoy, Lauriane Ledieu, Sophie Ricordel, Daniel Astrié, Bruno Tassin, Johnny Gasperi

**Affiliations:** a Univ Paris Est Créteil, LEESU, F-94010 Créteil, France; b Ecole des Ponts, LEESU, F-77455 Champs-sur-Marne, France; c GERS-LEE, Univ Gustave Eiffel, IFSTTAR, F-44344 Bouguenais, France; d Institut de Recherche en Sciences et Techniques de la Ville IRSTV, CNRS, 1 rue de la Noë, 44321 Nantes Cedex 3, France

**Keywords:** Macroplastics, Road runoff, Road traffic, Stormwater, TOP10 items, TSG-ML/OSPAR classification

## Abstract

Land-based sources of riverine macrolitter are now recognized as a major concern, but few field data on litter amount, composition and sources are available. This is especially the case for macrolitter hotspots like high frequented roadways that could generate large amount of macrolitter potentially reaching rivers. This dataset provides macrolitter amount and composition over one year from a retention pond collecting stormwater and carried macrolitter from a 800 m portion of a highly frequented roadway (around 90,000 vehicles per day). The typology of macrolitter was defined using the TSG-ML/OSPAR classifications. A total of 36,439 items in which 84% of plastics were individually counted, classified and weighted by category for a total mass of 88.5 kg (60% of plastics). Raw data are available in Mendeley Data (DOI:10.17632/t6ryv6crjd.4). Top 10 items represent 92% by count of the total with plastic fragments (31%), cigarette butts (18%), EPS fragments (17%) or foam packaging (11%) as most common items. Top 10 items represent 72% by mass of the total with plastic fragments (24%) and Cardboard (13%) as most common items, followed by foam packaging (6%), wood fragments (6%), industrial plastic sheets (5%), rubber fragments (4%) and EPS fragments (4%). More than 94% of plastic items are below 1.9 g/item. This dataset is related to the research paper *Amount, composition and sources of macrolitter from a highly frequented roadway*.

## Specifications Table

**Table:** 

Subject	Environmental Sciences, Pollution
Specific subject area	Plastic leakage into the environment: sources and pathways.
Type of data	Table Graph Figure
How the data were acquired	Hand collection of macrolitter in a retention pond Air-dried at the lab Manual sorting and visual identification of items according to TSG-ML/OSPAR classifications [1]. Each category of tiem was weighed. Computation into Microsoft Excel sheets. Graph and figures from Microsoft Excel and Adobe illustrator.
Data format	Analyzed Filtered
Description of data collection	Macrolitter were collected in a retention pond collecting stormwater from a 800 m portion of the South part of the Cheviré Bridge, Nantes, France. A one-year survey (10 samples) was conducted on the macrolitter conveyed by stormwater runoff in the retention pond. For each campaing, all visible macrolitter above 1 cm were collected and brought to the lab for sorting and identification. Road traffic and precipitation were also recorded for each campaign.
Data source location	• Institution: Gustave Eiffel University • City: Nantes • Country: France • Lat. 47.1849; Long. −1.6144 • Raw data: Mendeley Data; DOI:10.17632/t6ryv6crjd.4
Data accessibility	Repository name: Mendeley Data Data identification number: DOI: 10.17632/t6ryv6crjd.4 https://data.mendeley.com/datasets/t6ryv6crjd/4
Related research article	Related research paper: [2] L. Ledieu, R. Tramoy, S. Ricordel, D. Astrie, B. Tassin, et J. Gasperi, 2022. Amount, composition and sources of macrolitter from a highly frequented roadway. Environ. Pollut., vol. 303, p. 119145. https://doi.org/10.1016/j.envpol.2022.119145.

## Value of the Data

• Identified Macrolitter items, especially plastic debris, are scarce along roadsides in the peer-reviewed literature. This dataset participates to fill this knowledge gap with macrolitter collected on a highly frequented highway. Macrolitter amount were reported by count and by mass to facilitate emission estimates. In contrast to other data on macrolitter in the environment, macrolitter from the logistic sector (industrial sheeting, foam, and cardboard fragments) are commonly featuring in Top 10 items either by count or by mass.
• Macrolitter were characterized according to TSG-ML/OSPAR classifications to facilitate comparisons with other studies dealing with macrolitter leakage into the terrestrial and aquatic environment. Institutions, policy makers and researchers using this European classification and its future updates can benefit from those data.
• Exploring driving factors of the macrolitter accumulation over time was made possible thanks to road traffic data and other environmental data (wind, precipitation, temperature). Those data can be used on similar roads from which road traffic is known to estimate potential related litter and macroplastic emissions.

## 1 Data Description

Significant contributions from urban runoff to riverine macrolitter and plastic debris was already demonstrated [3], [4], [5]. Road runoff constitutes a potential non-point source of pollution as roadside ditches may connect land-based sources to waterway, but field data specifically dedicated to roadsides are scarce [6,7]. In this paper, an inventory of macrolitter from a portion of 800 m of a highly frequented highway in Nantes (France) is presented.

The dataset is made of 36,439 items >1 cm counted and classified according to TSG-ML/OSPAR, in which 84% of items were plastics (raw data in Mendeley Data; DOI:10.17632/t6ryv6crjd.4). Dry mass of each category was also reported for a total mass of 88.5 kg (60% of plastics), which is equivalent to 117.4 kg/yr/km or 42.8 kg/yr/ha.

**Fig. 1:**
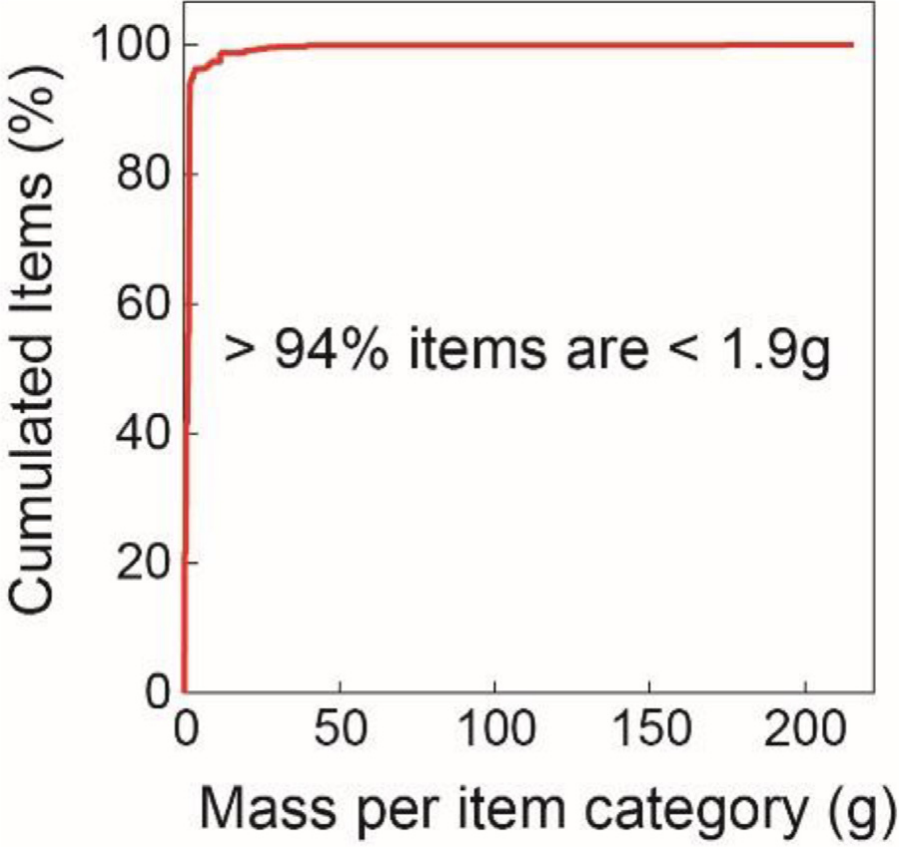
Mass distribution of plastic items only. The mass item per category corresponds to the average mass of items for a specific category, i.e. the number of items in a category divided by its mass, meaning the 30,777 plastic items were not individually weighed. Data are from Mendeley Data (DOI:10.17632/t6ryv6crjd.4).

Material types are reported in Table 1 by count and by mass together with precipitation amounts and road traffic for the 10 field campaigns. When focused on plastics, their mass distribution is shifted toward light weight specific items with a median value of 1.2 g/plastic and 94% < 1.9 g/plastic (n = 30,777; Fig. 1).

**Table 1:** Material types by count and mass of the ten field campaigns (C1 to C10) with associated precipitation (in mm) and cumulated road traffic. Mveh, Millions of vehicles.

Field campaigns	C1	C2	C3	C4	C5	C6	C7	C8	C9	C10	Ctot
**Start**	10/08/2020	08/09/2020	24/09/2020	20/10/2020	30/10/2020	02/12/2020	06/01/2021	26/01/2021	16/03/2021	06/05/2021	-
**End**	07/09/2020	23/09/2020	19/10/2020	29/10/2020	01/12/2020	05/01/2021	25/01/2021	15/03/2021	05/05/2021	29/07/2021	-
**Period (d)**	28	15	25	9	32	34	19	48	50	84	344
**Precipitation (mm)**	84.6	35.8	82.6	42.9	48.3	138.4	49.1	111.4	32.6	199.8	825.5
**Road traffic (Mveh)**	2.89	1.67	2.62	1.04	2.10	2.85	1.69	4.17	4.18	8.77	31.98
**Vehicles/d**	103,205	111,433	104,615	115,850	65,484	81,369	84,505	85,157	82,017	103,169	93,680.4

**Macrolitter by count and material type**											

**Plastics**	1,658	475	1,497	631	2,830	9,742	2,359	2,300	4,284	5,001	30,777
**%**	97.7	80.4	86.8	65.0	88.8	91.0	85.5	72.6	80.5	79.2	84.5
**Rubber**	5	9	16	3	11	16	35	31	42	66	234
**%**	0.3	1.5	0.9	0.3	0.3	0.1	1.3	1.0	0.8	1.0	0.6
**Textile**	0	17	22	6	11	25	18	20	37	76	232
**%**	0.0	2.9	1.3	0.6	0.3	0.2	0.7	0.6	0.7	1.2	0.6
**Paper, cardboard**	25	75	127	15	165	359	233	626	869	921	3,415
**%**	1.5	12.7	7.4	1.5	5.2	3.4	8.4	19.8	16.3	14.6	9.4
**Wood**	3	1	41	310	145	506	87	84	43	139	1,359
**%**	0.2	0.2	2.4	31.9	4.6	4.7	3.2	2.7	0.8	2.2	3.7
**Metal**	3	12	18	6	24	50	22	106	40	106	387
**%**	0.2	2.0	1.0	0.6	0.8	0.5	0.8	3.3	0.8	1.7	1.1
**Glass, ceramic**	2	1	4	0	0	1	5	0	8	9	30
**%**	0.1	0.2	0.2	0.0	0.0	0.0	0.2	0.0	0.2	0.1	0.1
**Other**	1	1	0	0	0	1	0	2	0	0	5
**%**	0.1	0.2	0.0	0.0	0.0	0.0	0.0	0.1	0.0	0.0	0.0
**Total**	**1,697**	**591**	**1,725**	**971**	**3,186**	**10,700**	**2,759**	**3,169**	**5,323**	**6,318**	**36,439**
**Macrolitter by mass (kg) and material type**											

**Plastics**	5.093	1.897	3.724	1.295	4.746	9.926	2.985	5.921	5.858	11.786	53.231
**%**	80.0	67.5	67.0	63.1	72.1	61.8	61.3	55.0	51.6	53.5	60.2
**Rubber**	0.120	0.110	0.177	0.025	0.208	1.164	0.490	0.607	0.944	0.443	4.289
**%**	1.9	3.4	3.2	1.2	3.2	7.2	10.1	5.6	8.3	2.0	4.8
**Textile**	0.470	0.176	0.375	0.028	0.234	1.410	0.214	0.245	0.536	1.841	5.528
**%**	7.4	5.5	6.7	1.4	3.5	8.8	4.4	2.3	4.7	8.4	6.2
**Paper, cardboard**	0.145	0.147	0.604	0.150	0.902	1.888	0.456	2.550	3.659	5.253	15.753
**%**	2.3	4.6	10.9	7.3	13.7	11.8	9.4	23.7	32.2	23.8	17.7
**Wood**	0.004	0.003	0.360	0.448	0.433	1.571	0.523	0.573	0.179	0.891	4.983
**%**	0.1	0.1	6.5	21.8	6.6	9.8	10.7	5.3	1.6	4.0	5.6
**Metal**	0.404	0.468	0.305	0.106	0.064	0.085	0.143	0.870	0.164	1.730	4.338
**%**	6.3	14.6	5.5	5.2	1.0	0.5	2.9	8.1	1.4	7.9	4.9
**Glass, ceramic**	0.040	0.003	0.017	0.000	0.000	0.011	0.056	0.000	0.022	0.091	0.241
**%**	0.6	0.1	0.3	0.0	0.0	0.1	1.2	0.0	0.2	0.4	0.3
**Other**	0.088	0.006	0.000	0.000	0.000	0.003	0.000	0.007	0.000	0.000	0.105
**%**	1.4	0.2	0.0	0.0	0.0	0.0	0.0	0.1	0.0	0.0	0.1
**Total**	**6.364**	**2.810**	**5.561**	**2.052**	**6.586**	**16.058**	**4.866**	**10.774**	**11.362**	**22.035**	**88.467**

**Fig. 2:**
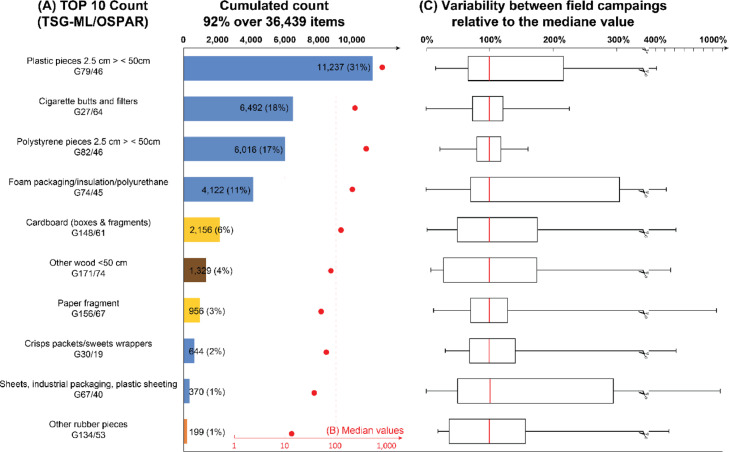
Top 10 macrolitter items collected in the retention pond of a highly frequented highway in Nantes, France. A, cumulated Top 10 items by count. Blue for plastics, yellow for cardboard and paper, brown for other wood (manufactured) and orange for rubber. B, median values between the ten field campaigns. C, variability in % relative to the median values (red bars) between the ten field campaigns. Lower and upper hinges represent the first and the third quartile and whiskers represent minimum and maximum values. Data are from Mendeley dataset (DOI:10.17632/t6ryv6crjd.4).

**Fig. 3:**
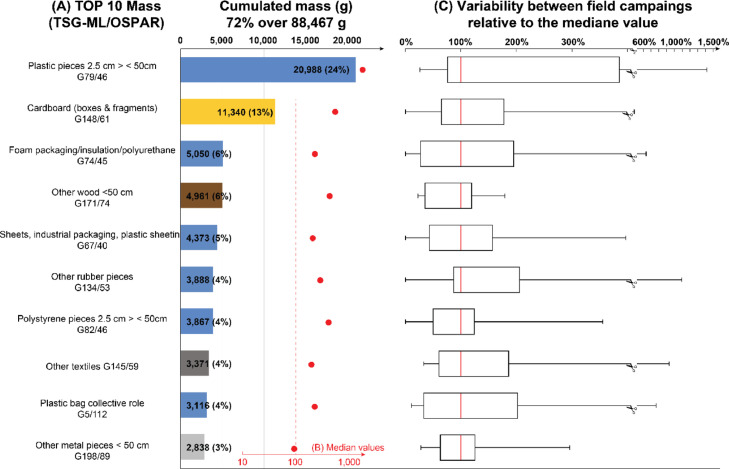
Top 10 macrolitter items collected in the retention pond of a highly frequented highway in Nantes, France. A, cumulated Top 10 items by mass. Blue for plastics, yellow for cardboard and paper, brown for wood, dark grey for clothing and textile and grey for metal. and orange for rubber. B, median values between the ten field campaigns. C, variability in % relative to the median values (red bars) between the ten field campaigns. Lower and upper hinges represent the first and the third quartile and whiskers represent minimum and maximum values. Data are from Mendeley dataset (DOI:10.17632/t6ryv6crjd.4).

Top 10 specific items by count and by mass based on aggregated data of all field campaigns are illustrated in Figs. 2 and 3, respectively. Top 10 items represent 92% by count of the total items (n = 36,439) and Top 10 items by mass represent 72% of the total mass, i.e. 88,467 g. Variability of abundances and masses is extremely high between the 10 campaigns with values spanning up to three orders of magnitude. Only the abundance distribution of cigarette butts and EPS fragments follows a normal distribution.

The most specific items featuring in both Top 10 are plastic fragments (31% by count and 24% by mass), EPS fragments (17% by count and 4% by count), foam packaging (11% by count and 6% by mass), Cardboards (6% by count and 13% by mass), wood fragments (4% by count and 6% by mass), industrial plastic sheets (1% by count and 5% by mass) and rubber fragments(1% by count and 4% by mass). Cigarette butts, paper fragments and sweet wrappers are only featuring in the Top 10 by count and represent respectively 18%, 3% and 2%. Other textiles, plastic bags and metal fragments are only featuring in the Top 10 by mass and represent respectively 4%, 4% and 3%.

Accumulation time, road traffic and rainfall are potential driving factors of the macrolitter accumulation in the retention pond. The 6^th^ field campaign corresponds to the Bella stormwater with high rainfall and high wind gusts. When field campaign 6 (C6) is excluded, macrolitter accumulation by count significantly correlates with the accumulation time and heavy vehicles traffic (Table 2). Macrolitter accumulation by mass also significantly correlates with the accumulation time and heavy vehicles traffic (Table 3). There are no significant correlations between macrolitter by count and rainfall.

**Table 2:** Spearman correlation (R) coefficient between main material types by count and time, traffic, Heavy (HV) vehicles (around 10% total traffic) and rainfall. Significant correlations (p-value < 0.05) are in bold.

	Correlation coefficient (Spearman, R)							
	C6 included				C6 excluded			
Items by count	Time	Traffic	HV vehicles	Rainfall	Time	Traffic	HV vehicles	Rainfall
All macrolitter	0.84	0.67	0.81	0.48	**0.90**	0.78	**0.90**	0.3
Plastics	0.79	0.64	0.76	0.46	**0.85**	0.73	**0.86**	0.27

**Table 3:** Spearman correlation (R) coefficient between main material types by mass and time, traffic, Heavy (HV) vehicles (around 10% total traffic) and rainfall. Significant correlations (p-value < 0.05) are in bold.

	Correlation coefficient (Spearman, R)							
	C6 included				C6 excluded			
Items by mass	Time	Traffic	HV vehicles	Rainfall	Time	Traffic	HV vehicles	Rainfall
All macrolitter	**0.96**	**0.88**	**0.93**	0.58	**1.00**	**0.95**	**1.00**	0.42
Plastics	**0.94**	**0.90**	**0.89**	0.70	**0.97**	**0.97**	**0.97**	0.58

## 2 Experimental Design, Materials and Methods

Macrolitter were collected in a retention pond collecting stormwater from a 800 m portion of the South part of the Cheviré Bridge (See Figure in Ledieu et al. [2]). A one-year survey was conducted on the macrolitter conveyed by stormwater runoff in the retention pond (Table 1). The Cheviré Bridge is in the western part of “Nantes Métropole” and is a part of its ring-road. It therefore constitutes a highly frequented highway over a length of 1,531 m. No pedestrians nor bikes may use this bridge, motorists are therefore the only potential input source of debris. During the studied period, an average of 93,680 ± 16,147 vehicles crossed that bridge each day in both directions (personal communication from DIRO – Direction Interdépartementale des Routes Ouest). Among these traffic levels, rates of heavy vehicles were relatively constant (9.9 ± 1.2%). The 800 m road portion investigated is divided into 2 × 3 lanes of traffic for a total surface of 20,639 m². Lateral gutters collect stormwater to a retention pond, south of the bridge. This pond offers a good opportunity to easily collect macrolitter. Rain amounts and wind speeds were measured at the Nantes-Atlantique airport station, 3 km from the Cheviré Bridge (https://prevision-meteo.ch/).

Ten field campaigns (C1 to C10) were performed over one year from the 10^th^ of August 2020 to the 29^th^ of July 2021. For each campaign, all macrolitter above 1 cm in the retention pond were collected by hands, air-dried at ambient air for days (at least one week) in the lab, characterized according to TSG-ML/OSPAR classifications [1] and weighed by category. The abundance of items was expressed by count and by dry mass. Plastic debris were considered as all artificial polymer materials, from parent codes G1 to G124 according to the TSG-ML classification. Raw data are available in Mendeley Data (DOI:10.17632/t6ryv6crjd.4).

Accumulation periods of macrolitter in the retention pond ranged between 9 and 84 days before sampling and associated precipitation amount ranged between 32.6 mm and 199.8 mm (Table 1). Road traffic ranged between 65,484 and 115,850 vehicles/d. The sample C5 partially corresponds to the second national lockdown relative to the COVID-19 pandemic (from October 30^th^ to December 15^th^, 2020), but levels of road traffic exhibited no significant differences with the other campaigns. The sample C6 integrates the Bella storm that occurred on December 27^th^ and 28^th^, 2020. During this storm, 20 mm of rain fell in one day and winds gusted up to 90 km/h (https://www.infoclimat.fr/).

## Ethics Statements

 

## CRediT Author Statement

**Tramoy Romain:** Conceptualization, Writing – original draft preparation, Data curation, Methodology, Illustration; **Lauriane Ledieu:** Conceptualization, Writing – original draft preparation, Data curation, Writing – review & editing; **Sophie Ricordel:** Data acquisition, Data curation; **Daniel Astrié:** Data acquisition, Data curation; **Bruno Tassin:** Validation, Supervision, Writing – reviewi & editing; **Johnny Gasperi:** Conceptualization, Methodology, Data curation, Writing – review & editing, Validation, Supervision.

## Declaration of Competing Interest

The authors declare that they have no known competing financial interests or personal relationships that could have appeared to influence the work reported in this paper.

## Data Availability

Macrolitter along a highly frequented roadway (Original data) (Mendeley Data).
